# Comparative efficacy of tandem autologous versus autologous followed by allogeneic hematopoietic cell transplantation in patients with newly diagnosed multiple myeloma: a systematic review and meta-analysis of randomized controlled trials

**DOI:** 10.1186/1756-8722-6-2

**Published:** 2013-01-04

**Authors:** Mohamed A Kharfan-Dabaja, Mehdi Hamadani, Tea Reljic, Taiga Nishihori, William Bensinger, Benjamin Djulbegovic, Ambuj Kumar

**Affiliations:** 1 Blood and Marrow Transplantation Program, H. Lee Moffitt Cancer Center/University of South Florida College of Medicine, Tampa, FL, USA; 2 Bone Marrow Transplantation Program, Department of Internal Medicine, Division of Hematology-Oncology, American University of Beirut Medical Center, Beirut, Lebanon; 3 Osborn Hematopoietic Malignancy and Transplantation Program, West Virginia University, Morgantown, WV, USA; 4 Center for Evidence-Based Medicine and Health Outcomes Research, University of South Florida, Morsani College of Medicine, 12901 Bruce B. Downs Boulevard, MDC 27, Tampa, FL, 33612, USA; 5 Department of Medicine, Division of Oncology, University of Washington School of Medicine and Fred Hutchinson Cancer Center, Seattle, WA, USA; 6 Department of Health Outcomes and Behavior, Moffitt Cancer Center, Tampa, FL, USA; 7 Department of Hematologic Malignancies, Moffitt Cancer Center, Tampa, FL, USA

**Keywords:** Autologous hematopoietic stem cell transplantation, Allogeneic hematopoietic stem cell transplantation, Multiple myeloma, Systematic review

## Abstract

**Background:**

Despite advances in understanding of clinical, genetic, and molecular aspects of multiple myeloma (MM) and availability of more effective therapies, MM remains incurable. The autologous-allogeneic (auto-allo) hematopoietic cell transplantation (HCT) strategy is based on combining cytoreduction from high-dose (chemo- or chemoradio)-therapy with adoptive immunotherapy. However, conflicting results have been reported when an auto-allo HCT approach is compared to tandem autologous (auto-auto) HCT. A previously published meta-analysis has been reported; however, it suffers from serious methodological flaws.

**Methods:**

A systematic search identified 152 publications, of which five studies (enrolling 1538 patients) met inclusion criteria. All studies eligible for inclusion utilized biologic randomization.

**Results:**

Assessing response rates by achievement of at least a very good partial response did not differ among the treatment arms [risk ratio (RR) (95% CI) = 0.97 (0.87-1.09), p = 0.66]; but complete remission was higher in the auto-allo HCT arm [RR = 1.65 (1.25-2.19), p = 0.0005]. Event-free survival did not differ between auto-allo HCT group versus auto-auto HCT group using per-protocol analysis [hazard ratio (HR) = 0.78 (0.58-1.05)), p = 0.11] or using intention-to-treat analysis [HR = 0.83 (0.60-1.15), p = 0.26]. Overall survival (OS) did not differ among these treatment arms whether analyzed on per-protocol [HR = 0.88 (0.33-2.35), p = 0.79], or by intention-to-treat [HR = 0.80 (0.48-1.32), p = 0.39] analysis. Non-relapse mortality (NRM) was significantly worse with auto-allo HCT [RR (95%CI) = 3.55 (2.17-5.80), p < 0.00001].

**Conclusion:**

Despite higher complete remission rates, there is no improvement in OS with auto-allo HCT; but this approach results in higher NRM in patients with newly diagnosed MM. At present, totality of evidence suggests that an auto-allo HCT approach for patients with newly diagnosed myeloma should not be offered outside the setting of a clinical trial.

## Background

The past two decades witnessed major advances in treatment of multiple myeloma (MM), including introduction of high-dose therapy (HDT) (chemotherapy or chemoradiotherapy), autologous hematopoietic cell transplantation (auto-HCT), and other effective therapies including immunomodulatory drugs or proteasome inhibitors, namely bortezomib [1-5]. These new chemotherapeutic agents when used in combinations, have led to improvement in survival and a higher frequency and better quality of response; but have not translated into cure of this disease [3,4].

The concept of ″total therapy″ treatment approach for patients with newly diagnosed MM, using multi-agent induction regimens, tandem auto-auto HCT, and post-transplantation maintenance resulted in progressive increase in proportion of patients achieving complete remission (CR) [6]. The Intergroupe Francophone du Myelome (IFM) demonstrated that tandem auto-auto HCT improves overall survival (OS) among patients with myeloma, particularly if a very good partial response (VGPR) is not achieved after undergoing the first auto-HCT [7]. A meta-analysis by our group showed that tandem auto-auto HCT versus single auto-HCT in previously untreated MM results in improved response rates, but not improved OS [8].

Badros et al. demonstrated the feasibility of offering reduced-intensity conditioning (RIC) allogeneic (allo)-HCT as a salvage strategy in 31 patients with relapsed MM [9]. Seventeen (55%) of 31 cases had received at least two auto-HCT and 17 (55%) had progressive disease at time of allografting [9]. Despite these adverse clinical features, 19 (61%) patients achieved CR or a near CR, with the 100-day and overall non-relapse mortality (NRM) of 10% and 29%, respectively [9]. This suggests a beneficial graft-versus-myeloma (GVM) effect mediated by alloreactive donor T-cells is capable of disease control, even in MM refractory to HDT. Gahrton et al. compared outcomes of patients who received allo-HCT for relapsed MM during 1983–1993 and 1994–1998 showing improvement in NRM and OS for patients allografted during the later time period [10]. The authors speculate that earlier time to allografting (10 months versus 14 months), for patients transplanted during the later time period, probably contributed to this beneficial effect [10]. Similar results were recently reported by Kumar et al., where 1 year OS post allo-HCT improved in three successive eras (1989–1994, 1995–2000, and 2001–2005) and increased interval between time of MM diagnosis and allografting was found to be an independent adverse prognostic factor for OS [11].

Combining benefits of cytoreductive-therapy from HDT and auto-HCT with adoptive immunotherapy (from allo-HCT) forms the basis of auto-allo HCT treatment strategy in patients with MM. Conflicting results, however, have been noted when an auto-allo HCT approach has been compared to an auto-auto HCT strategy. A recent systematic review on the same issue was performed by Armeson et al. [12] However, this systematic review is limited by inclusion of an inappropriate study, in our opinion. That is, this systematic review included the study by Garban et al. which was not a true randomized controlled trial but rather represents comparisons from two parallel trials (IFM99-03 and IFM99-04) that enrolled allograft and autograft recipients separately. Most importantly, the systematic review by Armeson et al. did not attempt to evaluate the methodological quality of included studies, which is the one of the key reasons to conduct a systematic review. Assessment of risk of bias in the systematic review process provides explanations on whether the observed findings are indeed the effect of the intervention or as a result of bias. Accordingly, we performed a systematic review of published studies comparing auto-auto HCT with auto-allo HCT in patients with newly diagnosed MM that addresses all the issues that were not addressed in the systematic review by Armeson et al.

## Results

**Figure 1:**
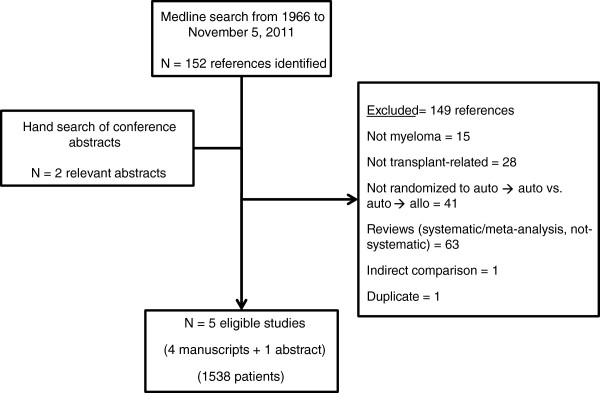
Flow-diagram depicting the identification and selection of eligible studies for inclusion in the systematic review.

Initial search yielded 152 references and 2 abstracts, of which 149 were excluded for various reasons as shown in Figure 1. Five studies (four full-manuscripts and one abstract) enrolling a total of 1538 patients were eligible for inclusion into this meta-analysis [13-17]. In one case [15], we identified a complementary publication [18] which provided longer follow-up on the originally published study. Additionally, we excluded one manuscript [19] because it was an indirect comparison (i.e. patients were enrolled separately into two parallel trials, IFM99-03 and IFM99-04, with different primary endpoints and subsequently compared to each other). Finally, we excluded one abstract, HOVON50/54, because patients on the control arm received only a single auto HCT [20].

### Patient, disease and treatment characteristics

Table 1 summarizes extracted data pertinent to patients′ disease and treatment characteristics. All studies allocated patients to auto-allo HCT if an HLA-matched sibling donor was available, except one [16] where matched volunteer unrelated donors were permitted. For patients undergoing tandem auto-auto HCT, high-dose melphalan 200 mg/m^2^ (MEL200) was the preferred regimen for the first autograft in three studies [13,14,16], melphalan dose ranging from 100 to 200 mg/m^2^ was used in one study [15], and melphalan dose ranging from 140 (with total body irradiation) to 200 mg/m^2^ was used in another study [17]. For the second autograft, MEL200 was the preferred regimen in two studies [13,16]. In the study by Bruno et al. patients were offered a dose of melphalan ranging from 100 to 200 mg/m^2^[15], whereas Rosiñol et al. allowed MEL200 or a combination of cyclophosphamide, etoposide, BCNU [17]. Moreover, Björkstrand et al. provided patients the option to undergo a second autograft using MEL200 or not to undergo a second autograft [14]. For the purpose of this meta-analysis, only patients who received a second autograft were included in analysis. 

**Table 1:** Characteristics of biologically randomized studies in tandem autologous versus autologous-allogeneic hematopoietic cell transplantation for patients with multiple myeloma

**Study**	**Publication type**	**Disease stage**	**Disease risk**	**Donor**	**Auto-allo regimen**	**Auto-auto regimen**
Björkstrand, 2011	Full text	DSS stage: I - 44 (12%) II - 55 (16%) III - 253 (72%)	Various	HLA matched sibling donor	MEL 200 mg/m2 → 2 Gy TBI + FLU	MEL 200 mg/m^2^ x 2 (or MEL 200 mg/m^2^ → no transplant (a second auto-HCT was optional)
Bruno, 2007/ Giaccone, 2011	Full text	DSS stage: II - 48 (30%) III - 114 (70%)	Various	HLA matched sibling donor	MEL 200 mg/m2 → 2 Gy TBI	MEL 100, 140 or 200 mg/m2 → MEL100, 140 or 200 mg/m2
Knop, 2009	Abstract	DSS stage: II and III	Limited to 13q-	HLA matched sibling or unrelated donors	MEL 200 mg/m2 → FLU-MEL 140 mg/m2 ± ATG	MEL 200 mg/m2 x 2
Krishnan & Pasquini, 2011	Full text	DSS stage: I-II - 201 (32%) III - 424 (68%)	Various	HLA matched sibling donor	MEL 200 mg/m2 → 2 Gy TBI	MEL 200 mg/m2 x 2
Rosiñol, 2008	Full text	ISS stage: I - 42 (40%) II - 48 (46%) III - 14 (14%)	Various	HLA matched sibling donor	MEL 200 mg/m2 or MEL 140 mg/m2 + TBI → FLU-MEL	MEL 200 mg/m2 or MEL 140 mg/m2 + TBI → MEL 200 mg/m2 or CBV

Abbreviations: *MEL*: melphalan; *FLU*: fludarabine; *CBV*: cyclophosphamide, etoposide, *BCNU*; *TBI*: total body irradiation; *DSS*: Durie-Salmon Staging System; *ISS*: International staging system.

For patients who received an auto-allo HCT approach, MEL200 was the preferred regimen for autografting in four studies [13-16]. RIC regimen of 2 Gy TBI was the preparative regimen in two studies [13,15]. Bjorkstrand et al. combined fludarabine with 2 Gy TBI [14], while the two remaining studies employed a RIC regimen with fludarabine/melphalan for allo-HCT [16,17]. No specific disease-risk eligibility criteria were required except in one study which limited enrollment to patients with deletion of chromosome 13q [16].

### Methodological quality

Methodological quality of included studies is summarized in Table 2. Briefly, all five studies utilized biologic randomization. Four studies reported data on prognostic factors and groups were balanced for presence of associated prognostic risk factors [13-15,17,18] while one study did not report data on prognostic factors [16]. None of the studies reported whether all consecutive patients were enrolled. Four studies had at least 1:2 ratio of auto-allo HCT to auto-auto HCT patients while one study [17] had a 1:3.4 ratio. None of the five studies reported blinding of any study personnel. Four studies [13-15,17,18] reported using the same reference time for assessing time dependent outcomes while one study [16] did not report a reference time. Three studies [13-15,18] reported outcomes according to intention-to-treat (ITT) and three studies [14,15,17,18] reported harms for patients treated per protocol. One study reported *a priori* expected difference, pre-specified α and β error, and sample size calculation [13].

**Table 2:** Methodological quality of biologically randomized studies in tandem autologous versus autologous-allogeneic hematopoietic cell transplantation for patients with multiple myeloma

	**Risk of bias**								**Risk of random error**		
**Study**	**Groups balanced on prognostic factors**	**All consecutive patients included**	**At least 1:2 ratio of auto-allo HCT versus auto-auto HCT**	**Description of withdrawals/ dropouts**	**Blinding of any study personnel**	**Same reference time used for both arms**	**Report ITT analysis of benefits**	**Report per protocol analysis of harms**	**A priori expected difference stated**	**α & β error pre-specified**	**Sample size calculations performed**
Björkstrand, 2011	yes	unclear	yes	no	no	yes	yes	yes	no	no	no
Giaccone, 2011	yes	unclear	yes	yes	no	yes	yes	yes	no	no	no
Knop, 2009	unclear	unclear	yes	no	no	unclear	unclear	unclear	no	no	no
Krishnan & Pasquini, 2011	yes	unclear	yes	yes	no	yes	yes	no	yes	yes	yes
Rosiñol, 2008	yes	unclear	no	yes	no	yes	no	yes	no	no	no

### Benefits

Summary of all evidence is presented in Table 3. 

**Table 3:** Summary of evidence for tandem autologous versus autologous-allogeneic hematopoietic cell transplantation in patients with multiple myeloma

**Quality assessment**			**No of patients**		**Effect**	
**No of studies**	**Design**	**Risk of bias**	**Auto-allo HCT**	**Auto-auto HCT**	**Relative (95% CI)**	**Absolute**
**Overall response rate**						
3	Biologically randomized trials	very serious^1,2^	248/275 (90.2%)	204/223 (91.5%)	RR 0.98 (0.92 to 1.05)	18 fewer per 1000 (from 73 fewer to 46 more)
**Complete response**						
5	Biologically randomized trials	very serious^1,2^	257/456 (56.4%)	254/674 (37.7%)	RR 1.65 (1.25 to 2.19)	245 more per 1000 (from 94 more to 448 more)
**At least very good partial response**						
1	Biologically randomized trials	very serious^1,2^	113/156 (72.4%)	272/366 (74.3%)	RR 0.97 (0.87 to 1.09)	22 fewer per 1000 (from 97 fewer to 67 more)
**Event-free survival (ITT)**						
3	Biologically randomized trials	serious^1^	414	815	HR 0.83 (0.60 to 1.15)	-
**Event-free survival (per-protocol)**						
4	Biologically randomized trials	very serious^1,2^	174	235	HR 0.78 (0.58 to 1.05)	-
**Overall survival (ITT)**						
3	Biologically randomized trials	serious^1^	414	815	HR 0.80 (0.48 to 1.32)	-
**Overall survival (per-protocol)**						
2	Biologically randomized trials	very serious^1,2^	83	131	HR 0.88 (0.33 to 2.35)	-
**Non-relapse mortality**						
4	Biologically randomized trials	serious^1^	50/363 (13.8%)	25/684 (3.7%)	RR 3.55 (2.17 to 5.80)	93 more per 1000 (from 43 more to 175 more)
**Grade II-IV GVHD**^3^						
4	Biologically randomized trials	serious^1^	126/485 (26%)	-	Proportion 28.26 (20.65 to 36.55)	-
**Chronic GVHD**^3^						
4	Biologically randomized trials	serious^1^	206/356 (57.9%)	-	Proportion 60.69 (50.65 to 70.29)	-

**1** Selective outcome reporting; **2** Per-protocol reporting of benefits; **3** GVHD: graft-versus-host disease.

#### Response rates

Response data was reported per protocol in four studies and one study reported all outcomes according to both ITT and per protocol [14]. Two studies [14,17] used European Bone Marrow Transplantation (EBMT) criteria [21] for response assessment; one study [13] used International Uniform Response (IUR) Criteria [22], while the (more stringent CR and PR) criteria used by Bruno et al. was described [15]. One study did not report how response was assessed [16].

**Figure 2:**
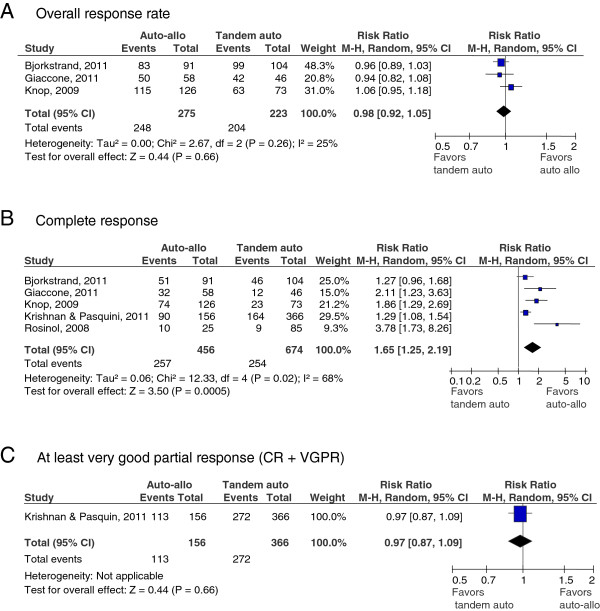
**A through 2C: Forest plot for response rates. Overall (A), complete (B) or at least very good partial response (C).** The summary estimate (risk ratio) from individual studies is indicated by rectangles with lines representing the 95% confidence intervals (CIs). The summary pooled estimate from all studies is represented by the diamond and the stretch of the diamond indicates the corresponding 95% CI.

As illustrated in Figure 2 A-C, the pooled results (three studies [14,16,18] with 498 patients) showed no significant difference in overall response rate (ORR) between auto-allo HCT versus auto-auto HCT [risk ratio (RR) (95% confidence interval [CI]) = 0.98 (0.92-1.05), p = 0.66]. There was low heterogeneity between pooled studies for the outcome of ORR (I^2^ =25%). The pooled results for CR from five studies [13,14,16-18] (1130 patients) showed a statistically significant benefit in treatment with auto-allo HCT over auto-auto HCT [RR (95% CI) = 1.65 (1.25-2.19), p ≤ 0.001]. However, there was statistically significant heterogeneity among pooled studies (I^2^ =68%). Results for at least VGPR (one study [13] enrolling 522 patients) showed no significant difference between either treatment strategy [RR (95% CI) = 0.97 (0.87-1.09), p = 0.66].

#### Event-free survival

**Figure 3:**
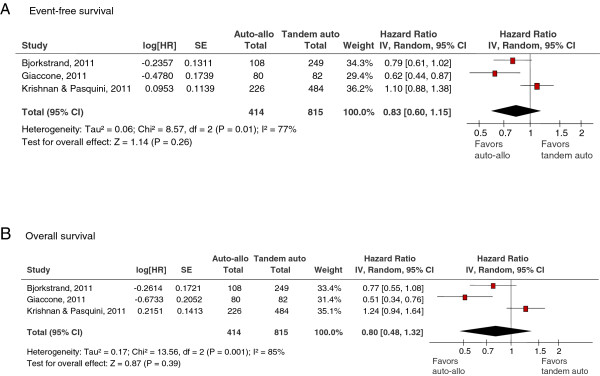
**A and 3B: Forest plot for event-free survival according to intent-to-treat analysis (A) and overall survival (B).** The summary estimate (hazard ratio) from individual studies is indicated by rectangles with lines representing the 95% confidence intervals (CIs). The summary pooled estimate from all studies is represented by the diamond and the stretch of the diamond indicates the corresponding 95% CI.

None of the studies reported definitions for event-free survival (EFS) or progression-free survival (PFS). For this analysis, EFS data was used when reported, otherwise PFS was substituted. As presented in Figure 3A, the pooled results from three studies [13,14,18] (1229 patients) which reported EFS according to ITT showed no significant difference between treatment with auto-allo HCT versus auto-auto HCT [hazard ratio (HR) (95% CI) = 0.83 (0.60-1.15), p = 0.26]. Pooled results for three studies [14,17,18] (409 patients) which reported EFS per protocol also showed no significant difference in treatment with auto-allo HCT [HR (95% CI) = 0.78 (0.58-1.05), p = 0.11] compared with auto-auto HCT. Heterogeneity among studies included in ITT analysis was significant (I^2^ =77%) while heterogeneity in per-protocol analysis was moderate (I^2^ =32%).

#### Overall survival

As illustrated in Figure 3B, the pooled results (three studies [13,14,18] enrolling 1229 patients) for OS according to ITT showed no significant difference in treatment with auto-allo HCT versus auto-auto HCT [HR (95% CI) = 0.80 (0.48-1.32), p = 0.39]. The pooled results from two studies [17,18] (214 patients) which reported OS per-protocol also showed no significant difference between the two treatment modalities [HR (95% CI) = 0.88 (0.33-2.35), p = 0.79]. There was a statistically significant heterogeneity whether OS was analyzed according to ITT (I^2^ =85%) or per-protocol (I^2^ =77%).

### Harms

#### Non-relapse mortality

**Figure 4:**
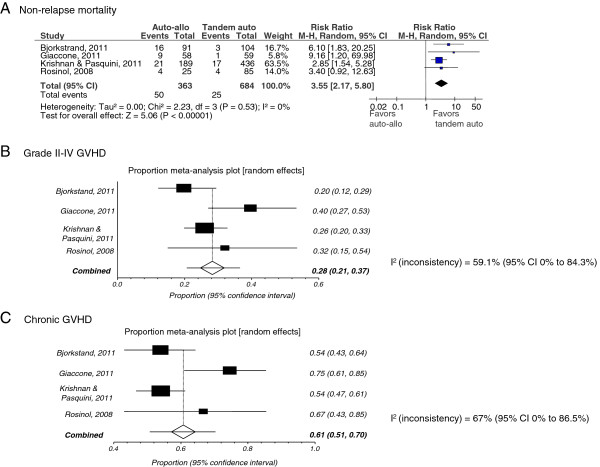
**A through 4C: Forest plot for non-relapse mortality (A), grade II-IV graft versus-host disease (B) and chronic graft versus-host disease (C).** The summary estimate (risk ratio/proportions) from individual studies is indicated by rectangles with lines representing the 95% confidence intervals (CIs). The summary pooled estimate from all studies is represented by the diamond and the stretch of the diamond indicates the corresponding 95% CI. For the proportional meta-analysis the diamond represents the pooled summary estimates and the 95% CI is indicated by the line.

Pooled results from four studies [13,14,17,18] (1047 patients) showed NRM was significantly worse with an auto-allo HCT approach [RR (95% CI) = 3.55 (2.17-5.80), p < 0.00001] compared to auto-auto HCT (Figure 4A). There was no heterogeneity among included studies (I^2^ =0%).

#### Graft-versus-host disease

Incidence of any acute graft-versus-host disease (GVHD) was reported in one study [14] (91 patients) and the proportion of patients undergoing auto-allo HCT with any GVHD was 30.77% (95% CI 21.51-41.32). Incidence of grade II-IV GVHD was reported in four studies [13,14,17,18] (363 patients), and the pooled proportion of patients undergoing auto-allo HCT with grade II-IV GVHD was 28.26% (95% CI 20.65-36.55; see Figure 4B). Heterogeneity among studies reporting grade II-IV GVHD was borderline (I^2^ = 59%). Incidence of chronic GVHD was reported in four studies [13,14,17,18] (356 patients), and the pooled proportion of patients undergoing auto-allo HCT with chronic GVHD was 60.69% (95% CI 50.65-70.29; Figure 4C). Heterogeneity among studies reporting chronic GVHD was significant (I^2^ = 67%).

### Sensitivity analysis/subgroup analysis

To assess robustness of the pooled results and explore possible reasons for heterogeneity, additional sensitivity and subgroup analyses were performed (see Table 4). To evaluate robustness of response outcomes, sensitivity analysis was performed according to response criteria (EBMT [21], IUR [22], non-EBMT/IUR [15], and not reported). There was no significant difference in ORR or CR regardless of criteria used. Sensitivity analysis for primary outcome of OS was performed according to all elements of risk of bias. Significant differences in pooled results were only detected when per protocol analysis of OS in a study (104 patients) which included at least 1:2 ratio of auto-allo HCT versus auto-auto HCT [HR (95% CI) = 0.55 (0.32-0.94) p = 0.03] was compared with per protocol analysis of OS in a study (110) which did not include at least 1:2 ratio of auto-allo HCT versus auto-auto HCT [HR (95% CI) = 1.51 (0.70-3.27) p = 0.30]. Sensitivity analysis according to risk of bias did not explain reasons for observed heterogeneity of primary outcome. For risk of random error, while one study [13] (710 patients) which reported sample size calculations showed no difference in OS [HR (95% CI) = 1.24 (0.94-1.64), p = 0.13], the pooled results from two studies [14,18] (519 patients) which did not report sample size calculations showed a significant OS benefit with use of auto-allo HCT versus auto-auto HCT [HR (95% CI) = 0.64 (0.43-0.95), p = 0.03]. There was statistically significant heterogeneity between the two studies which did not report sample size calculations (I^2^ = 58%). 

**Table 4:** Sensitivity analyses by response criteria and significant elements of quality

**Subgroup**	**Outcome**	**Studies**	**Patients**	**HR or RR**	**95% CI**	**Heterogeneity**	**Test of Interaction**
Tool used to capture response							
EBMT criteria	ORR (per-protocol)	1	195	0.96	(0.96–1.04)	NA	0.26
Non-EBMT/IUR criteria		1	104	0.94	(0.82–1.08)	NA	
Criteria not reported		1	199	1.06	(0.95–1.18)	NA	
EBMT criteria	CR (per-protocol)	2	305	2.05	(0.71–5.98)	85%	0.12
IUR criteria		1	522	1.29	(1.08–1.54)	NA	
Non-EBMT/IUR criteria		1	104	2.11	(1.23–3.63)	NA	
Criteria not reported		1	199	1.86	(1.29–1.54)	NA	
At least 1:2 ratio of auto-allo HCT versus auto-auto HCT							
Yes	OS (per-protocol)	1	104	0.55	(0.32–0.94)	NA	0.04
No		1	110	1.51	(0.70–3.27)	NA	
Description of withdrawals/dropouts							
Yes	OS (ITT)	2	872	0.81	(0.34–1.92)	92%	0.93
No		1	357	0.77	(0.55–1.08)	NA	
Report calculation of sample size							
Yes	OS (ITT)	1	710	1.24	(0.94–1.64)	NA	0.007
No		2	519	0.64	(0.43–0.95)	58%	

Abbreviations: *EBMT*: European Group for Blood and Marrow Transplantation response criteria; *IUR*: International Uniform Response criteria; *ORR*: overall response rate; *CR*: complete response; *OS*: overall survival; *ITT*: intention-to-treat; *NA*: not applicable.

## Discussion

Auto-HCT has been regarded as the standard of care for younger myeloma patients [1,23]. However, much controversy exists about the role and timing of allo-HCT in newly diagnosed MM. Our meta-analysis indicates despite higher CR rates following an auto-allo HCT approach, there is no apparent improvement in OS, whether comparative analysis is performed as per-protocol or on ITT basis. This is likely explained by significantly higher NRM associated with RIC allo-HCT versus a second auto-HCT [RR (95% CI) = 3.55 (2.17-5.80), p < 0.00001]. Accordingly, further improvements in the auto-allo HCT approach will require strategies to significantly reduce NRM and augment anti-myeloma effects. Not surprising, significant cause of NRM in the auto-allo HCT arm resulted from development of acute and/or chronic GVHD in these patients. For instance, in the study by Krishnan et al. eight (13%) of 60 deaths were attributed to GVHD [13]. Similarly, in the study by Rosiñol et al., three (75%) of four cases of NRM were from complications of acute GVHD [17]. This suggests that future treatment strategies aimed at exploiting GVM effects, in auto-allo HCT approach, should avoid exacerbating GVHD at all costs. It is noteworthy that OS benefit with an auto-allo HCT approach is limited to studies using 2 Gy TBI-based conditioning regimens [14,15], which has led to speculation [14] that the lack of survival benefit in other studies might relate to use of more intense conditioning which is associated with increased regimen-related toxicity and mortality in those studies [16,17]. It is important to indicate the largest trial by Krishnan et al. [13] used 2 Gy TBI conditioning but was also subject to referral bias, and to date has not reported any survival benefit.

Conceptually, auto-allo HCT approach combines the advantage of cytoreduction from HDT from the first autograft with the benefit of adoptive immunotherapy resulting from the donor T cell alloreactivity. Notwithstanding, in the study by Krishnan et al. 22 (37%) of 60 deaths in the auto-allo HCT arm were still due to MM [13]. As a result, future strategies should aim at achieving deeper remissions, namely molecular remissions, or a state of minimal residual disease, prior to moving forward with allografting. This might entail evaluating novel potent therapies during the peri-allografting phase. Moreover, designing more effective regimens for allo-HCT, beyond 2 Gy TBI, is likely necessary to improve outcomes.

In regards to using auto-auto HCT as the control arm for comparison in these studies, one could argue that this approach is not yet considered the standard of care in all patients with newly diagnosed MM. In fact, outcomes from various studies comparing single auto-HCT versus tandem auto-auto approach have been discrepant [7,24,25] and a published meta-analysis failed to show OS benefit with tandem autografts [8].

A major limitation of all studies comparing auto-auto HCT to auto-allo HCT is lack of detailed information about disease/genetic risk stratification. Only one study limited accrual to patients with deletion 13q detectable by FISH [16]. However prognostic significance of 13q deletion detected by FISH as opposed to conventional cytogenetics remains questionable [26]. Whether an auto-allo HCT approach might be beneficial for high-risk MM is not known, and should be further assessed in future trials [27-29]. We were not able to assess if auto-allo HCT approach might be beneficial for high risk myeloma patients as included studies did not report results according to risk categories for all outcomes. An individual patient data meta-analysis would be suitable to answer this question. Furthermore, the results are prone to outcome reporting bias as only three studies reported OS data according to ITT [13,14,18] and another study reported data using per-protocol analysis only [17].

The findings are also somewhat different from the systematic review by Armeson et al. as we excluded a manuscript published by Garban et al. because it aimed at comparing two parallel trials (IFM99-03 and the IFM99-04) which enrolled allograft and autograft recipients separately [12]. The objectives of the IFM99-03 trial were to evaluate the feasibility and NRM of RIC allografting [19], whereas the primary end point of IFM99-04 was to compare CR rates achieved after the second auto HCT (with or without anti–IL-6 monoclonal antibody BE-8). Additionally, we excluded a cohort of high-risk patients reported by a study by Krishnan et al. because the original aim of this study was to assess progression-free survival among standard-risk patients [13]. The investigators reported only partial data on a smaller cohort of high-risk patients.

## Conclusions

Efforts at identifying particular subgroups of patients with MM, based on prognostic clinical, biological, cytogenetic and genetic risk factors, which are likely to benefit from an auto-allo HCT approach is necessary to help refine the role of this approach in MM. At the present, totality of evidence suggests that an auto-allo HCT approach for patients with newly diagnosed myeloma should not be offered outside the setting of a clinical trial.

## Methods

### Identification of eligible studies

Any completed study in newly diagnosed MM patients comparing auto-auto HCT versus auto-allo HCT was eligible for inclusion in this systematic review. Studies which did not utilize biologic randomization or were indirect comparisons of tandem auto-auto HCT versus auto-allo HCT were excluded.

A systematic search of MEDLINE database thru Nov 5, 2011, and pertinent conference proceedings (American Society of Hematology, American Society of Clinical Oncology, European Hematology Association, American Society for Blood and Marrow Transplantation, and EBMT Group) was conducted to identify relevant publications. The following search strategy was used: (″Multiple Myeloma″ [Mesh] AND ″Transplantation, Autologous″ [Mesh] AND ″Transplantation, Homologous″ [Mesh]). No search limits were applied based on language.

### Study selection and data extraction

Two authors (M.A.K-D and M.H.) appraised the list of references and selected studies in consultation with other authors (T.R. and A.K.). Disagreements were resolved by consensus. Dual data extraction on clinical outcomes, treatment benefits and harms, and methodological quality of included studies was undertaken. Since biologic randomization is not similar to traditional randomized controlled trials, not all elements of risk of bias were applicable. For methodological quality, we extracted data on the following elements: comparability of two groups on all aspects except the intervention (e.g. disease stage, age, gender, etc.), enrollment of consecutive patients, enrollment of patients in auto-allo and auto-auto group in at least 1:2 ratio, description of withdrawals and dropouts (if any), blinding of study personnel and who was blinded (e.g. data collectors, outcome assessors etc.), comparability of reference time used for time-dependent outcomes between treatment groups and analysis according to ITT principle for benefits and per-protocol for adverse events. Clinical outcomes analyzed included: response rates (ORR, CR and VGPR), OS, EFS, NRM and GVHD. For purposes of this review, OS was considered the primary outcome; response rates, EFS, NRM and GVHD were considered secondary outcomes.

### Statistical analysis

Dichotomous data were summarized using RR based on number of events and total number of patients and pooled under random-effects model. For time-to event data, HR and 95% CI were extracted when reported. When authors did not report time-to-event estimates, we extracted data from publication using methods described by Tierney et al. [30]. Time-to-event data were pooled using generic inverse variance under random-effects model. For analysis of proportional data, methods by Stuart et al. [31] were used to transform proportions into a quantity according to Freeman-Tukey variant of the arcsine square root transformed proportion [31]. Pooled proportion was calculated as a back-transform of the weighted mean of the transformed proportions, using random-effects model [31]. All data are reported with 95% CI. Calculation of the I^2^ statistic was used to test for heterogeneity. An I^2^ > 50% was considered statistically significant heterogeneity [32]. To assess robustness of the pooled results and explore possible reasons for heterogeneity, additional sensitivity analyses/subgroup analyses were performed according to publication type, patient and disease characteristics, and methodological quality of included studies (risk of bias and random error). All analysis were performed using RevMan 5.1 [33] and StatsDirect [34] software. This work is reported according to the PRISMA guidelines [35].

## Abbreviations

MM: Multiple myeloma; HDT: High-dose therapy; auto: Autologous; HCT: Hematopoietic cell transplantation; CR: Complete remission; IFM: Intergroupe Francophone du Myelome; OS: Overall survival; auto-auto: Tandem autologous; VGPR: Very good partial response; RIC: Reduced-intensity conditioning; auto-allo: Autologous followed by allogeneic; NRM: Non-relapse mortality; GVM: Graft-versus-myeloma; HOVON: Hemato-Oncologie voor Volwassenen Nederland; ITT: Intention-to-treat; EBMT: European Bone Marrow Transplantation Group; ORR: Overall response rate; EFS: Event-free survival; GVHD: Graft-versus-host disease; RR: Risk ratio; HR: Hazard ratio; CI: Confidence intervals; PRISMA: Preferred Reporting Items for Systematic Reviews and Meta-Analyses; IUR: International Uniform Response Criteria.

## Competing interests

All authors have no competing interests that may be relevant to the submitted work.

## Authors’ contributions

MAKD, MH, BD, and AK were responsible for conception and design of this review. MAKD, MH, TR and AK performed the literature search and study selection. MAKD, MH, TR, and AK collected all data. MAKD, MH, TR, WB, BD, and AK contributed to the data analysis and interpretation of results. MAKD, MH and AK drafted the manuscript, MAKD, MH, TR, WB, BD, and AK revised the manuscript critically for important intellectual content. MAKD, MH, TR, WB, BD, and AK approved the final version of the manuscript to be published. All authors read and approved the final manuscript.

## Previous presentation

Parts of this manuscript have been presented as an oral presentation at the Annual Meeting of the European Group for Blood and Marrow Transplantation 2012 (Abstract 520).

## References

[B1] Attal M Harousseau JL Stoppa AM Sotto JJ Fuzibet JG Rossi JF Casassus P Maisonneuve H Facon T Ifrah N A prospective, randomized trial of autologous bone marrow transplantation and chemotherapy in multiple myeloma. Intergroupe francais du myelome N Engl J Med 1996 335 91 97 10.1056/NEJM199607113350204 8649495

[B2] Kumar SK Rajkumar SV Dispenzieri A Lacy MQ Hayman SR Buadi FK Zeldenrust SR Dingli D Russell SJ Lust JA Improved survival in multiple myeloma and the impact of novel therapies Blood 2008 111 2516 2520 10.1182/blood-2007-10-116129 17975015 PMC2254544

[B3] Richardson PG Barlogie B Berenson J Singhal S Jagannath S Irwin D Rajkumar SV Srkalovic G Alsina M Alexanian R A phase 2 study of bortezomib in relapsed, refractory myeloma N Engl J Med 2003 348 2609 2617 10.1056/NEJMoa030288 12826635

[B4] Richardson PG Schlossman RL Weller E Hideshima T Mitsiades C Davies F LeBlanc R Catley LP Doss D Kelly K Immunomodulatory drug CC-5013 overcomes drug resistance and is well tolerated in patients with relapsed multiple myeloma Blood 2002 100 3063 3067 10.1182/blood-2002-03-0996 12384400

[B5] Singhal S Mehta J Desikan R Ayers D Roberson P Eddlemon P Munshi N Anaissie E Wilson C Dhodapkar M Antitumor activity of thalidomide in refractory multiple myeloma N Engl J Med 1999 341 1565 1571 10.1056/NEJM199911183412102 10564685

[B6] Barlogie B Jagannath S Desikan KR Mattox S Vesole D Siegel D Tricot G Munshi N Fassas A Singhal S Total therapy with tandem transplants for newly diagnosed multiple myeloma Blood 1999 93 55 65 9864146

[B7] Attal M Harousseau JL Facon T Guilhot F Doyen C Fuzibet JG Monconduit M Hulin C Caillot D Bouabdallah R Single versus double autologous stem-cell transplantation for multiple myeloma N Engl J Med 2003 349 2495 2502 10.1056/NEJMoa032290 14695409

[B8] Kumar A Kharfan-Dabaja MA Glasmacher A Djulbegovic B Tandem versus single autologous hematopoietic cell transplantation for the treatment of multiple myeloma: a systematic review and meta-analysis J Natl Cancer Inst 2009 101 100 106 10.1093/jnci/djn439 19141779

[B9] Badros A Barlogie B Siegel E Cottler-Fox M Zangari M Fassas A Morris C Anaissie E Van Rhee F Tricot G Improved outcome of allogeneic transplantation in high-risk multiple myeloma patients after nonmyeloablative conditioning J Clin Oncol 2002 20 1295 1303 10.1200/JCO.20.5.1295 11870172

[B10] Gahrton G Svensson H Cavo M Apperly J Bacigalupo A Björkstrand B Blade J Cornelissen J de Laurenzi A Facon T Progress in allogenic bone marrow and peripheral blood stem cell transplantation for multiple myeloma: a comparison between transplants performed 1983–93 and 1994–8 at European group for blood and marrow transplantation centres Br J Haematol 2001 113 209 216 10.1046/j.1365-2141.2001.02726.x 11360893

[B11] Kumar S Zhang MJ Li P Dispenzieri A Milone GA Lonial S Krishnan A Maiolino A Wirk B Weiss B Trends in allogeneic stem cell transplantation for multiple myeloma: a CIBMTR analysis Blood 2011 118 1979 1988 10.1182/blood-2011-02-337329 21690560 PMC3158724

[B12] Armeson KE Hill EG Costa LJ Tandem autologous vs autologous plus reduced intensity allogeneic transplantation in the upfront management of multiple myeloma: meta-analysis of trials with biological assignment Bone Marrow Transplant 2012 22964593 10.1038/bmt.2012.173 PMC5975377 22964593

[B13] Krishnan A Pasquini MC Logan B Stadtmauer EA Vesole DH Alyea E 3rd Antin JH Comenzo R Goodman S Hari P Autologous haemopoietic stem-cell transplantation followed by allogeneic or autologous haemopoietic stem-cell transplantation in patients with multiple myeloma (BMT CTN 0102): a phase 3 biological assignment trial Lancet Oncol 2011 12 1195 1203 10.1016/S1470-2045(11)70243-1 21962393 PMC3611089

[B14] Björkstrand B Iacobelli S Hegenbart U Gruber A Greinix H Volin L Narni F Musto P Beksac M Bosi A Tandem autologous/reduced-intensity conditioning allogeneic stem-cell transplantation versus autologous transplantation in myeloma: long-term follow-up J Clin Oncol 2011 29 3016 3022 10.1200/JCO.2010.32.7312 21730266

[B15] Bruno B Rotta M Patriarca F Mordini N Allione B Carnevale-Schianca F Giaccone L Sorasio R Omede P Baldi I A comparison of allografting with autografting for newly diagnosed myeloma N Engl J Med 2007 356 1110 1120 10.1056/NEJMoa065464 17360989

[B16] Knop S Liebisch P Hebart H Holler E Engelhardt M Bargou R Metzner B Peest D Aulitzky W Wandt H Allogeneic stem cell transplant versus tandem high-dose melphalan for front-line treatment of deletion 13q14 myeloma – an interim analysis of the german DSMM V trial Blood 2011 114 51

[B17] Rosiñol L Perez-Simon JA Sureda A de la Rubia J de Arriba F Lahuerta JJ Gonzalez JD Diaz-Mediavilla J Hernandez B Garcia-Frade J A prospective PETHEMA study of tandem autologous transplantation versus autograft followed by reduced-intensity conditioning allogeneic transplantation in newly diagnosed multiple myeloma Blood 2008 112 3591 3593 10.1182/blood-2008-02-141598 18612103

[B18] Giaccone L Storer B Patriarca F Rotta M Sorasio R Allione B Carnevale-Schianca F Festuccia M Brunello L Omede P Long-term follow-up of a comparison of nonmyeloablative allografting with autografting for newly diagnosed myeloma Blood 2011 117 6721 6727 10.1182/blood-2011-03-339945 21490341 PMC3251223

[B19] Garban F Attal M Michallet M Hulin C Bourhis JH Yakoub-Agha I Lamy T Marit G Maloisel F Berthou C Prospective comparison of autologous stem cell transplantation followed by dose-reduced allograft (IFM99-03 trial) with tandem autologous stem cell transplantation (IFM99-04 trial) in high-risk de novo multiple myeloma Blood 2006 107 3474 3480 10.1182/blood-2005-09-3869 16397129

[B20] Lokhorst H Sonneveld P Holt B Oers M Raymakers R Zweegman S Minnema M Zijlmans M Donor versus no donor analysis of newly diagnosed myeloma patients included in the HOVON 50/54 study Blood 2008 112 461 10.1182/blood-2007-09-077438 22442350

[B21] Blade J Samson D Reece D Apperley J Björkstrand B Gahrton G Gertz M Giralt S Jagannath S Vesole D Criteria for evaluating disease response and progression in patients with multiple myeloma treated by high-dose therapy and haemopoietic stem cell transplantation. Myeloma subcommittee of the EBMT. European group for blood and marrow transplant Br J Haematol 1998 102 1115 1123 10.1046/j.1365-2141.1998.00930.x 9753033

[B22] Durie BG Harousseau JL Miguel JS Blade J Barlogie B Anderson K Gertz M Dimopoulos M Westin J Sonneveld P International uniform response criteria for multiple myeloma Leukemia 2006 20 1467 1473 10.1038/sj.leu.2404284 16855634

[B23] Child JA Morgan GJ Davies FE Owen RG Bell SE Hawkins K Brown J Drayson MT Selby PJ High-dose chemotherapy with hematopoietic stem-cell rescue for multiple myeloma N Engl J Med 2003 348 1875 1883 10.1056/NEJMoa022340 12736280

[B24] Cavo M Tosi P Zamagni E Cellini C Tacchetti P Patriarca F Di Raimondo F Volpe E Ronconi S Cangini D Prospective, randomized study of single compared with double autologous stem-cell transplantation for multiple myeloma: bologna 96 clinical study J Clin Oncol 2007 25 2434 2441 10.1200/JCO.2006.10.2509 17485707

[B25] Sonneveld P van der Holt B Segeren CM Vellenga E Croockewit AJ Verhoe GE Cornelissen JJ Schaafsma MR van Oers MH Wijermans PW Intermediate-dose melphalan compared with myeloablative treatment in multiple myeloma: long-term follow-up of the dutch cooperative group HOVON 24 trial Haematologica 2007 92 928 935 10.3324/haematol.11168 17606443

[B26] Avet-Loiseau H Attal M Moreau P Charbonnel C Garban F Hulin C Leyvraz S Michallet M Yakoub-Agha I Garderet L Genetic abnormalities and survival in multiple myeloma: the experience of the intergroupe francophone du myelome Blood 2007 109 3489 3495 10.1182/blood-2006-08-040410 17209057

[B27] Dispenzieri A Is there a future for auto-allo HSCT in multiple myeloma? Lancet Oncol 2011 12 1176 1177 10.1016/S1470-2045(11)70258-3 21962392

[B28] Dispenzieri A Rajkumar SV Gertz MA Fonseca R Lacy MQ Bergsagel PL Kyle RA Greipp PR Witzig TE Reeder CB Treatment of newly diagnosed multiple myeloma based on mayo stratification of myeloma and risk-adapted therapy (mSMART): consensus statement Mayo Clin Proc 2007 82 323 341 17352369 10.4065/82.3.323

[B29] Kumar SK Mikhael JR Buadi FK Dingli D Dispenzieri A Fonseca R Gertz MA Greipp PR Hayman SR Kyle RA Management of newly diagnosed symptomatic multiple myeloma: updated mayo stratification of myeloma and risk-adapted therapy (mSMART) consensus guidelines Mayo Clin Proc 2009 84 1095 1110 10.4065/mcp.2009.0603 19955246 PMC2787395

[B30] Tierney JF Stewart LA Ghersi D Burdett S Sydes MR Practical methods for incorporating summary time-to-event data into meta-analysis Trials 2007 8 16 10.1186/1745-6215-8-16 17555582 PMC1920534

[B31] Stuart A Ord K Kendall′s advanced theory of statistics 1994 Wiley

[B32] Higgins JPT Green S Cochrane Handbook for Systematic Reviews of Interventions, Version 5.1.0 edition 2011 The Cochrane Collaboration

[B33] RevMan: Review Manager The Cochrane Collaboration-available on the world wide web51st http://www.cochrane.org/cochrane/revman.htm

[B34] StatsDirect Ltd StatsDirect statistical software 2008 England: Stats Direct Ltd http://www.statsdirect.com

[B35] Liberati A Altman DG Tetzlaff J Mulrow C Gotzsche PC Ioannidis JP Clarke M Devereaux PJ Kleijnen J Moher D The PRISMA statement for reporting systematic reviews and meta-analyses of studies that evaluate health care interventions: explanation and elaboration J Clin Epidemiol 2009 62 e1 34 10.1016/j.jclinepi.2009.06.006 19631507

